# Atypical Emergency Department Presentation of Acute Motor and Sensory Axonal Neuropathy: A Case Report

**DOI:** 10.5811/cpcem.65644

**Published:** 2026-10-13

**Authors:** Shadman Kabir, Austin Rahman, Sitha Konopack

**Affiliations:** University of Florida College of Medicine, Gainesville and UF Health Shands Hospital, Department of Emergency Medicine, Gainesville, Florida

**Keywords:** acute motor and sensory axonal neuropathy (AMSAN), Guillain-Barré syndrome, emergency department, diagnostic error, case report

## Abstract

**Introduction:**

Acute motor and sensory axonal neuropathy is a rare, severe axonal variant of Guillain–Barré syndrome characterized by rapidly progressive motor and sensory dysfunction. Early recognition in the emergency department (ED) is challenging because patients may initially present with subtle neurologic findings, preserved reflexes, and normal cerebrospinal fluid (CSF) studies.

**Case Report:**

A 29-year-old woman with a history of generalized anxiety disorder presented to the ED with progressive distal numbness and mild weakness, with weakness being her chief complaint. Neurologic examination demonstrated preserved reflexes without focal deficits, and she was noted to be anxious. Given the reassuring examination, the patient was discharged with strict return precautions, including instructions to seek immediate reevaluation for worsening weakness, numbness, or progression of neurologic symptoms. She returned 48 hours later with rapidly worsening ascending weakness. The patient’s clinical progression prompted neurology consultation and hospital admission despite initially reassuring examination findings. During a five-day hospitalization, electrodiagnostic testing and clinical findings established the diagnosis of acute motor and sensory axonal neuropathy.

This case illustrates how acute motor and sensory axonal neuropathy may initially mimic benign or functional conditions, particularly in patients with underlying anxiety, preserved reflexes, and normal early CSF findings. In the ED, anchoring bias and premature diagnostic closure may delay recognition of serious neurologic disease. Serial neurologic examinations, maintaining a broad differential diagnosis, and early neurology consultation are critical when evaluating patients with progressive neurologic symptoms.

**Conclusion:**

Acute motor and sensory axonal neuropathy may present atypically during the early stages of disease, and initial examination and laboratory findings may be misleading. Clinical progression, rather than isolated examination or diagnostic findings, should guide disposition and prompt reassessment to facilitate timely diagnosis and treatment, with the potential to improve patient outcomes.

## INTRODUCTION

Guillain-Barré syndrome is an acute immune-mediated polyneuropathy classically characterized by ascending weakness and sensory deficits.^1^,^6^ It is most commonly associated with demyelination of peripheral nerves, although axonal variants such as acute motor and sensory axonal neuropathy (AMSAN) occur and are often associated with more severe disease.^2^,^3^,^6^ Guillain-Barré syndrome is frequently preceded by a gastrointestinal or respiratory infection.^2^,^6^

Early recognition in the emergency department (ED) remains challenging because patients may initially lack the classic findings of areflexia, progressive ascending paralysis, and albuminocytologic dissociation.^4^,^6^,^7^ This case highlights the importance of serial neurologic examinations, maintaining a broad differential diagnosis, and early neurology consultation when evaluating patients with progressive neurologic symptoms suggestive of Guillain-Barré syndrome despite initially reassuring findings.

## CASE REPORT

A 29-year-old woman with a history of generalized anxiety disorder presented to a community satellite ED with three days of progressive bilateral hand and foot numbness. Her medical history was notable for generalized anxiety disorder. Surgical history included tonsillectomy. Family history was noncontributory except for a cousin with multiple sclerosis. She denied tobacco, alcohol, or illicit drug use. Medications included vitamin D supplementation; she was not taking her prescribed bupropion. She reported a latex allergy (rash).

Her symptoms began approximately one week after a viral upper respiratory infection, initially as three days of intermittent hand paresthesias that progressed to persistent distal sensory deficits bilaterally in her upper arms/hands. She subsequently developed neck and shoulder discomfort and headache, along with early functional impairment, including difficulty lifting her toddler. She denied chest pain, shortness of breath, nausea, vomiting, and bowel or bladder dysfunction.

A focused neurologic examination demonstrated intact strength (5/5) in bilateral upper and lower extremities except for mild weakness (4/5) in finger abduction and adduction bilaterally. Reflexes were 2+ throughout, and Hoffman sign was negative bilaterally. Sensory examination revealed decreased light touch, pinprick, and vibration only in the hands (distal digits, palms) and feet (mid-foot) without a clear dermatomal distribution. Temperature sensation was preserved. Cranial nerve examination was normal, gait was steady, and no focal neurologic deficits were identified. Based on pertinent information at the time of presentation a broad differential diagnosis was considered, which included cervical myelopathy, inflammatory neuropathy including Guillain-Barré syndrome, metabolic derangements, and less likely central nervous system pathology. Many of the differentials had a low pretest probability based on presentation and duration of symptoms. Electrolyte abnormality was ruled out based on objective lab markers.

Laboratory evaluation, including complete blood count, basic metabolic panel, thyroid-stimulating hormone, magnesium, phosphorus, ionized calcium, urinalysis, and urine pregnancy test, were within normal limits. Computed tomography (CT) of the head and imaging of the cervical spine were unremarkable. The presence of bilateral upper distal symptoms following a recent viral illness raised concern for early Guillain-Barré syndrome. She received intravenous fluids, magnesium, nonsteroidal anti-inflammatory drugs, and vitamin supplementation without improvement. Due to diagnostic uncertainty and limited resources, she was transferred to a tertiary care ED for further evaluation and specialty consultation.

*CPC-EM Capsule*

What do we already know about this clinical entity?

*Acute motor and sensory axonal neuropathy (AMSAN) is a rare Guillain-Barré syndrome variant that may initially lack classic findings, delaying diagnosis.*

What makes this presentation of disease reportable?

*AMSAN presented with preserved reflexes, normal cerebrospinal fluid findings, and subtle upper extremity deficits, leading to a reassuring emergency evaluation.*

What is the major learning point?

*Serial neurologic examinations and recognition of clinical progression are essential when patients have persistent neurologic symptoms despite reassuring findings.*

How might this improve emergency medicine practice?

*Recognizing atypical Guillain-Barré syndrome presentations and avoiding anchoring bias can improve emergency department decision-making and timely treatment.*

At the tertiary care ED, her symptoms persisted, but reflexes remained preserved. Negative inspiratory force measured −40 cm H_2_O, above commonly used thresholds for ventilatory support but sufficiently reduced to warrant serial respiratory monitoring (reference range, −60 to −100 cm H_2_O). No other studies were pursued at the tertiary care center; however, neurology was consulted. Given preserved reflexes, stable respiratory parameters, and unremarkable diagnostic studies, Guillain-Barré syndrome was considered less likely. In conjunction with neurology, the ED team determined the patient was appropriate for discharge with strict return precautions, which included worsening of numbness or increased weakness in her extremities. A rheumatology and neurology referral was provided to the patient for further workup in the outpatient settin

She returned to the tertiary ED 48 hours later with worsening symptoms. These included progressive distal weakness (difficulty manipulating zippers and dropping objects), new generalized weakness, and ascending numbness in a stocking-glove distribution. Repeat neurologic examination on this visit showed the following:

- Right upper extremity: Deltoid, bicep, and triceps strength. 5/5; wrist extension, 4/5; finger abduction and finger extension, 3/5; finger flexion, 5/5 L
- Left upper extremity: Deltoid, biceps, and. triceps strength, 5/5; wrist extension, 5/5; finger flexion, 5/5; finger abduction and finger extension, 3/5

Lower extremities were 5/5 bilaterally. She demonstrated normal reflexes, unchanged from prior presentation.

Repeat noncontrast CT of the head and cervical spine showed no acute findings. A lumbar puncture was performed in the ED based on concern for Guillain-Barré syndrome, which showed CSF studies within normal limits (glucose, protein, cell count, clear colorless appearance). Given concern for rapidly evolving neuromuscular disease, magnetic resonance imaging of the brain and cervical spine were performed, which showed benign findings. After consultation with neurology, she was admitted to the neurology service. Corticosteroids were initiated while inflammatory myelopathy remained in the differential diagnosis.

During hospitalization, serial examinations demonstrated progression to symmetric distal greater than proximal weakness, areflexia in both upper and lower extremities, and persistent sensory deficits in her upper extremities (bilateral arms and hands). Repeat laboratory studies remained within normal limits. Serial respiratory monitoring showed negative inspiratory force values ranging from −40 to −52 cm H_2_O. On hospital day 3, electromyography and nerve conduction studies demonstrated a severe axonal sensorimotor polyneuropathy consistent with AMSAN, a variant of Guillain-Barré syndrome. Following electrodiagnostic confirmation, corticosteroids were discontinued and intravenous immunoglobulin was initiated. The patient demonstrated improvement in strength while maintaining stable respiratory function without the need for ventilatory support. She was discharged with persistent but improved distal weakness and referred for outpatient neuromuscular follow-up and rehabilitation.

## DISCUSSION

Acute motor and sensory axonal neuropathy is a rare axonal variant of Guillain-Barré syndrome characterized by immune-mediated injury to motor and sensory axons, often resulting in more severe clinical outcomes than demyelinating forms.^3^ Early recognition in the ED is notoriously difficult; as seen in this case, patients may initially present with subtle neurologic deficits while maintaining preserved deep tendon reflexes and normal CSF studies.^7^

Diagnostic frameworks emphasize that early Guillain-Barré syndrome frequently lacks the classic triad of progressive ascending weakness, sensory symptoms, and areflexia.^4^ Guillain-Barré syndrome encompasses multiple clinical variants, further contributing to diagnostic uncertainty. For example, Miller Fisher syndrome presents with ophthalmoplegia and ataxia,^14^ while the pharyngeal-cervical-brachial variant involves bulbar and upper extremity weakness rather than the classic ascending pattern.^15^ Unlike acute inflammatory demyelinating polyradiculoneuropathy, the demyelinating form of Guillain-Barré syndrome, AMSAN primarily involves axonal injury, which is associated with more severe weakness, slower recovery, and greater residual disability. Early clinical findings, however, may overlap substantially with other Guillain–Barré syndrome variants, making distinction difficult before electrodiagnostic testing. These variants underscore the clinical heterogeneity of Guillain-Barré syndrome and demonstrate why atypical presentations must remain in the differential diagnosis, even when traditional markers are absent.^2^,^4^

In this case, the patient’s history of generalized anxiety disorder potentially contributed to diagnostic anchoring—a cognitive bias where clinicians rely too heavily on initial information or psychiatric history rather than performing a careful neurologic reassessment.^13^ Awareness of such biases is essential to avoid premature diagnostic closure, especially since objective abnormalities in CSF and electrodiagnostic studies often lag behind clinical symptom onset.^2^,^7^ Recent literature highlights this variability, reinforcing the importance of prioritizing the clinical trajectory and serial examinations over single time-point assessments.^7^,^10^

This case underscores the importance of strict return precautions. Emergency physicians are responsible for the disposition of every patient they treat. In this instance, the medical team appropriately transferred the patient to a tertiary center for neurologic evaluation before her symptoms worsened. While clinicians might consider a lumbar puncture during the initial visit, normal CSF findings early in the course of Guillain-Barré syndrome would not have altered the immediate management. Albuminocytologic dissociation develops in only approximately half of patients during the first week of illness; therefore, a normal CSF profile should not exclude Guillain-Barré syndrome early in its course.

Ultimately, providing clear return instructions enabled the patient to seek timely follow-up care as her condition evolved. Emergency physicians must prioritize emphasizing these precautions during the discharge process.

## CONCLUSION

This case illustrates that early acute motor and sensory axonal neuropathy may not meet traditional diagnostic expectations despite evolving neuromuscular disease. In such patients, progression over time may be a more reliable diagnostic indicator than isolated examination findings. Beyond its low incidence, the clinical complexity of this case stemmed from an atypical presentation involving symptoms primarily in the upper extremities, preserved reflexes, and unremarkable CSF. These subtle findings complicated early diagnostic assessment and contributed to initially low clinical suspicion for Guillain-Barré syndrome.

The initial stages of Guillain-Barré syndrome frequently lack traditional diagnostic hallmarks, particularly within the first week of symptom onset, which complicates early identification. Consequently, this report stresses the necessity of performing serial neurologic assessments, obtaining prompt neurology consultation, and maintaining a high index of suspicion for Guillain-Barré syndrome even when initial workups are reassuring. The presence of progressive weakness warrants meticulous reassessment and careful disposition planning, regardless of preserved reflexes or normal CSF parameters.

For the emergency physician, this clinical scenario emphasizes the critical need to mitigate anchoring bias and recognize that unconventional presentations of Guillain-Barré syndrome may appear deceptively benign. Early recognition, close reassessment, and timely initiation of immunotherapy remain critical to reducing morbidity and preventing respiratory compromise in patients with Guillain-Barré syndrome.
